# Early emergence of neuroendocrine prostate cancer during triplet therapy for high-volume metastatic castration-sensitive prostate cancer

**DOI:** 10.1007/s13691-025-00812-8

**Published:** 2025-10-09

**Authors:** Yuichiro Nakamura, Hajime Fukushima, Hiroki Kurata, Junki Harada, Kyohei Araki, Kensuke Mitsunari, Tomohiro Matsuo, Kojiro Ohba, Yasushi Mochizuki, Ryoichi Imamura

**Affiliations:** Department of Urology, Nagasaki University Graduate School of Biomedical Sciences, 1-7-1 Sakamoto, Nagasaki, 852-8501 Japan

**Keywords:** Neuroendocrine prostate cancer, Metastatic castration-sensitive prostate cancer, Triplet therapy, Metastatic lesion biopsy

## Abstract

This case highlights the potential for early emergence of neuroendocrine differentiation in patients with metastatic castration-sensitive prostate cancer undergoing triplet therapy with androgen deprivation therapy, darolutamide, and docetaxel, even with marked prostate-specific antigen (PSA) suppression. In this patient, although some lesions initially responded to treatment, new metastases and radiological progression were observed early after triplet therapy was initiated. These atypical progression patterns raised the suspicion of neuroendocrine prostate cancer (NEPC), confirmed by percutaneous biopsy of an enlarged right external iliac lymph node. This enabled the timely modification of the treatment strategy. Furthermore, retrospective immunohistochemical reevaluation of the diagnostic prostate biopsy specimen obtained at the referring hospital using only four cores revealed focal neuroendocrine differentiation within the poorly differentiated Gleason pattern 5 areas, suggesting that de novo NEPC features may have been present at the time of diagnosis. As triplet therapy has become more widespread, the incidence of NEPC may increase. Clinicians should maintain a high level of vigilance for discordant PSA progression and consider early biopsy of metastatic lesions to ensure accurate diagnosis and appropriate therapeutic decision-making.

## Introduction

Androgen deprivation therapy (ADT) combined with androgen receptor signaling inhibitors (ARSIs), also known as ARSI-based doublet therapy, is the standard initial treatment for metastatic castration-sensitive prostate cancer (mCSPC). Recently, the addition of docetaxel triplet therapy has gained widespread use in clinical practice. However, treatment strategies following triplet therapy remain unclear, and the increasing use of ARSIs has raised concerns regarding the emergence of treatment-related neuroendocrine prostate cancer (t-NEPC). Here, we report a rare case of early NEPC development during triplet therapy, despite marked PSA suppression. This case highlights the importance of early biopsy and histopathological assessment of atypical radiographic progression during treatment.

## Case report

**Fig. 1:**
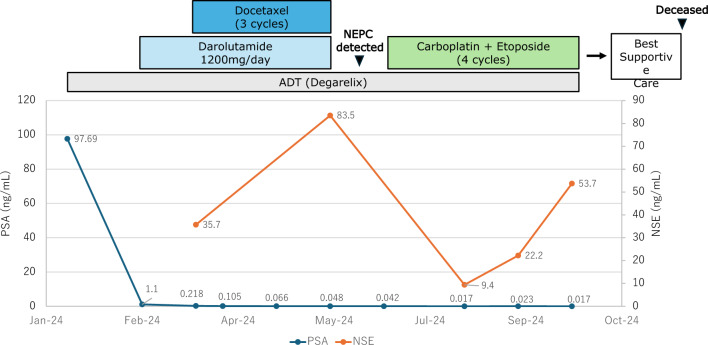
Clinical course of the patient. Prostate-specific antigen (PSA) and neuron-specific enolase (NSE) levels were plotted along a timeline of therapeutic interventions

**Fig. 2:**
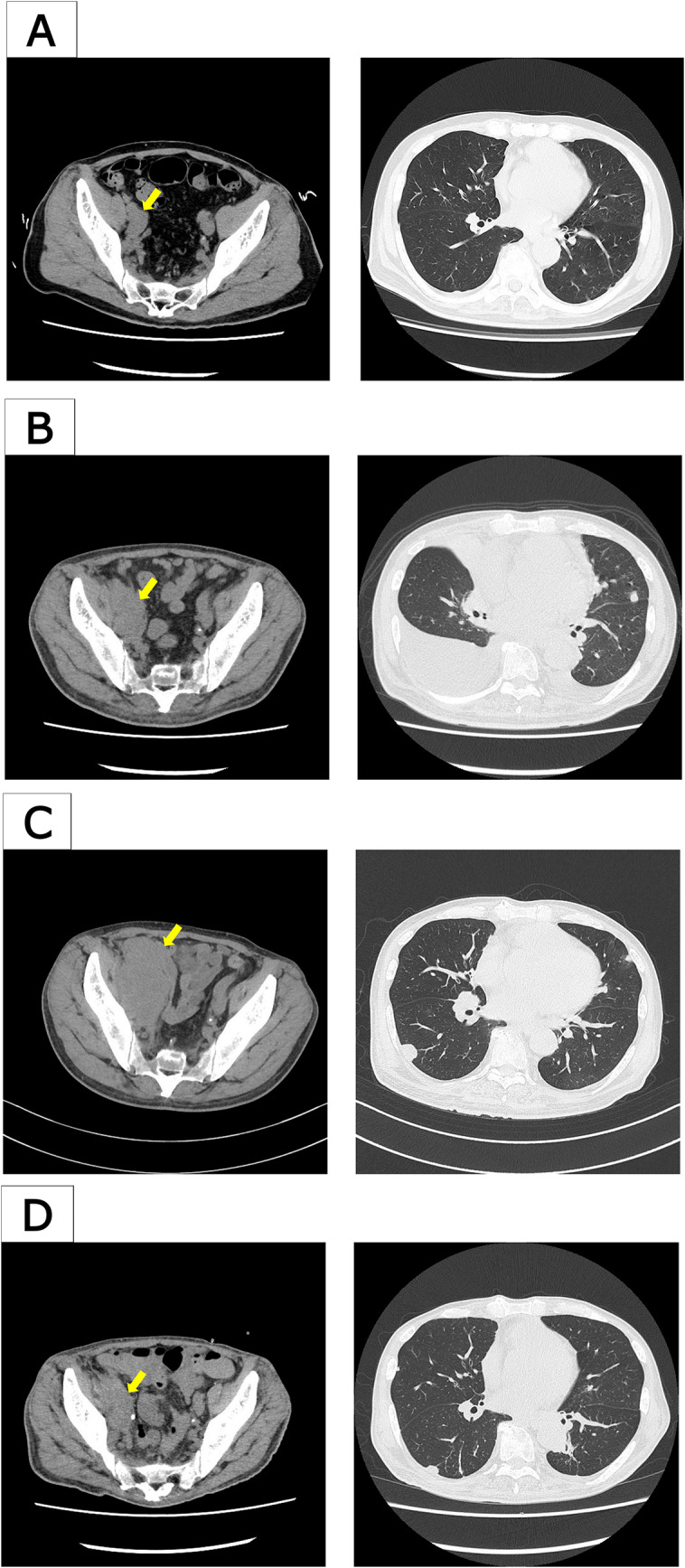
CT images before and after treatment modification. **A** Enlargement of the right external iliac lymph node without pulmonary metastases before initiation of triplet therapy. **B** Right external iliac lymph-node metastasis and appearance of pulmonary metastases after starting triplet therapy but before docetaxel administration. **C** After completion of three cycles of docetaxel (end of triplet therapy), showing progression with enlargement of the same lymph-node and new pulmonary metastases. **D** After two cycles of carboplatin plus etoposide (CBDCA + VP-16), partial regression of the lymph nodes and pulmonary lesions was observed

**Fig. 3:**
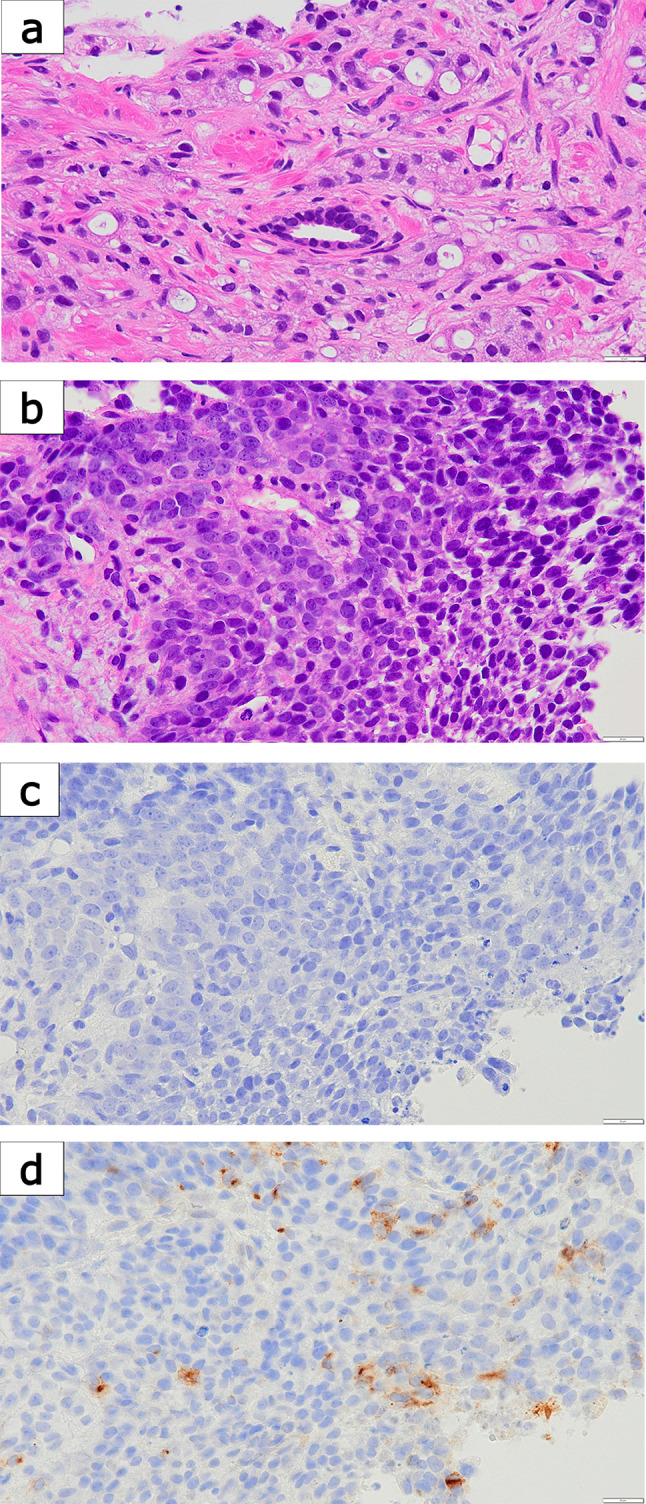
Histopathological and immunohistochemical findings. **a** Prostate biopsy (Gleason pattern 5) showing poorly differentiated adenocarcinoma without glandular structures (H&E, × 40). **b** Lymph-node metastasis showing poorly differentiated carcinoma with neuroendocrine morphology (H&E, × 40). **c** PSA immunostaining demonstrating complete negativity in the lymph-node specimen (× 40). **d** Synaptophysin immunostaining showing partial positivity in the lymph-node specimen (× 40)

A 61-year-old man presented with lower back pain and initially visited an orthopedic clinic, where imaging studies suggested metastatic bone tumors. His prostate-specific antigen (PSA) level increased to 97.69 ng/mL (Fig. 1). Computed tomography (CT) and magnetic resonance imaging (MRI) revealed multiple bone and lymph-node metastases, suggestive of prostate cancer (Fig. 2A). The clinical stage at diagnosis was cT2cN1M1b, with diffuse bone metastases involving nearly the entire axial skeleton, as confirmed by MRI and bone scintigraphy. No liver metastases were detected at diagnosis or during the subsequent clinical course. The patient was subsequently referred to the urology department at another hospital. The transrectal prostate biopsy performed in this study consisted of only four cores, which is less than the usual number. Biopsy revealed adenocarcinoma with the highest Gleason score of 4 + 5. The patient was diagnosed with high-volume mCSPC and referred to our department for treatment. Triplet treatment was initiated as the first-line therapy, beginning with ADT using degarelix. One month after treatment initiation, PSA decreased to 1.10 ng/mL. Darolutamide was added (Fig. 1). Two months after initiation (just before starting docetaxel), the PSA level further decreased to 0.218 ng/mL, and prostate shrinkage was observed on CT (Fig. 1). The primary prostate lesion remained reduced in size without evidence of regrowth throughout the course of therapy. However, enlargement of multiple lymph nodes and multiple new pulmonary metastases were observed, with neuron-specific enolase (NSE) elevated to 35.7 ng/mL (Fig. 2B). Docetaxel (75 mg/m^2^) combined with prednisolone (10 mg/day) was administered. After one docetaxel cycle, the PSA level decreased to 0.066 ng/mL, and CT revealed shrinkage of the pulmonary metastases. However, lymph nodes continued to increase in size. Owing to grade 4 neutropenia, the docetaxel dosage was reduced to 70 mg/m^2^ from the second cycle onward. After three cycles, the PSA level remained low at 0.048 ng/mL; however, CT revealed progression of lymph-node metastases and regrowth of previously shrinking pulmonary metastases (Fig. 2C). NSE levels also increased to 83.5 ng/mL (Fig. 1). Despite the early stages of triplet therapy, these findings indicated disease progression, and percutaneous needle biopsy of an enlarged right external iliac lymph node was performed. Histopathological examination revealed predominantly poorly differentiated-to-undifferentiated carcinoma with neuroendocrine morphology, consistent with NEPC (Fig. 3). A minor component retained features reminiscent of adenocarcinoma, characterized by clear cytoplasm and round nuclei but lacking glandular structures. Immunohistochemically, AE1/AE3 was positive, CK7 and CK20 were negative (supporting prostatic origin), and AMACR and androgen receptor (AR) were partially positive, whereas PSA was only faintly positive. Neuroendocrine differentiation was confirmed by positivity for synaptophysin (partial), CD56, and INSM1, whereas chromogranin A was negative.

Owing to compression of the right ureter by the enlarged lymph node, which caused renal dysfunction, a ureteral stent was placed. The treatment was changed to carboplatin (AUC 5) plus etoposide. After two cycles of this regimen, NSE decreased markedly to 9.4 ng/m. CT showed a significant reduction in lymph-node and pulmonary metastases (Fig. 2D). However, after four cycles, NSE levels increased again to 53.9 ng/mL, and mild progression of some lesions was observed (Fig. 1). Following the completion of four cycles, the patient developed febrile neutropenia complicated by septic shock, requiring intensive care unit admission. Therefore, chemotherapy was discontinued after four cycles.

Comprehensive genomic profiling (CGP) (FoundationOne CDx) of the metastatic biopsy specimen revealed APC loss, APC rearrangement, ARID1A mutation, FAS loss, PTEN loss, and RB1 loss. The microsatellite status was stable (MS-stable), and the tumor mutation burden was low (1 mutation/Mb), with no actionable targets identified for personalized therapy. The patient subsequently received the best supportive care and died approximately 2 months after the cessation of chemotherapy. Retrospective reevaluation of the initial prostate biopsy revealed distinct findings according to histological grade. In the Gleason 4 component, AMACR and AR were positive, PSA was weakly positive, NSE was negative, chromogranin A showed faint non-specific staining, and synaptophysin, CD56, and INSM1 were all negative. In contrast, the Gleason 5 component showed PSA negativity, partial AR positivity, and positivity for NSE, synaptophysin, CD56, and INSM1. These findings suggested that focal neuroendocrine differentiation was already present at diagnosis. The results of the immunohistochemical staining are summarized in Table 1.

**Table 1:** Immunohistochemical staining results of the prostate biopsy and lymph-node biopsy

Marker	Prostate biopsy (Gleason 4, higher differentiation)	Prostate biopsy (Gleason 5, poorly differentiated)	Lymph-node biopsy (adenocarcinoma-like component)	Lymph-node biopsy (poorly differentiated NEPC)
AE1/AE3	Not tested	Not tested	Positive	Positive
CK7	Not tested	Not tested	Negative	Negative
CK20	Not tested	Not tested	Negative	Negative
AMACR	Positive	Weak/negative	Partial positive	Negative
PSA	Weakly positive	Negative	Faint positive	Negative
AR	Positive	Partial positive	Partial positive	Negative
NSE	Negative	Positive	Not tested	Positive
Chromogranin A	Weak, non-specific positivity	Focal positive	Negative	Negative
Synaptophysin	Negative	Positive	Partial positive	Partial positive
CD56	Negative	Positive	Positive	Positive
INSM1	Negative	Positive	Positive	Positive

## Discussion

Triplet therapy, which consists of ADT, docetaxel, and ARSIs, has recently emerged as a standard first-line treatment option for patients with high-volume mCSPC endorsed by the National Comprehensive Cancer Network (NCCN) Guidelines (Version 1.2026) [1]. Although clinical trials have demonstrated its efficacy, real-world data remain limited, particularly regarding long-term outcomes and post-progression treatment strategies. However, recent observational studies, including a Japanese multicenter analysis, have begun to report real-world outcomes of triplet therapy in Asian populations [2]. Despite these developments, optimal therapeutic sequencing following the progression to metastatic castration-resistant prostate cancer (mCRPC) after triplet therapy has not been clearly established, highlighting the need for further clinical insights.

In this case, triplet therapy initially led to a marked reduction in PSA levels and a partial radiographic response in some metastatic lesions. Despite these biochemical improvements, early imaging studies have revealed disease progression, including newly developed pulmonary and lymph-node metastases. This discordance between PSA suppression and radiographic progression raised the suspicion of NEPC, prompting a percutaneous biopsy of an enlarged right external iliac lymph node. Histopathological examination revealed a poorly differentiated adenocarcinoma with neuroendocrine features, confirming the diagnosis of NEPC. Furthermore, retrospective immunohistochemical reevaluation of the initial diagnostic prostate biopsy, performed at the referring hospital with limited sampling (only four cores), revealed focal positivity for Chromogranin A, synaptophysin, and CD56, and negativity for PSA in the Gleason pattern 5 component. These findings suggest that de novo NEPC may have been present from the outset but initially remained undetected.

In this case, immunohistochemistry of the initial biopsy already demonstrated focal neuroendocrine differentiation in the poorly differentiated Gleason 5 component, whereas the Gleason 4 component retained AR and PSA expression without neuroendocrine marker positivity. These findings suggest that de novo NEPC features were present from the onset, despite limited sampling at diagnosis. The subsequent lymph-node biopsy confirmed progression, with most tumor cells showing complete loss of AR and PSA and strong expression of neuroendocrine markers, whereas a minor component retained adenocarcinoma-like morphology and faint PSA positivity. This coexistence supports the interpretation of de novo NEPC with mixed histology rather than pure t-NEPC.

Although t-NEPC cannot be entirely excluded—particularly since ARSIs and docetaxel have been implicated in promoting lineage plasticity and dedifferentiation [3, 4]—we consider de novo NEPC to be the more plausible explanation in this case. If t-NEPC were the main driver, the appropriateness of upfront intensified therapy such as triplet therapy could be questioned. Nevertheless, the rapid clinical progression despite PSA suppression highlights the importance of early biopsy and histological reassessment in patients exhibiting atypical disease evolution during intensified therapy.

NEPC is a rare histological subtype of prostate cancer that accounts for only 0.2–1% of newly diagnosed de novo NEPC cases [5, 6]. De novo NEPC is associated with a highly aggressive clinical course; approximately 64% of patients present with distant metastases at diagnosis, and the median overall survival (OS) is approximately 10 months [7]. Neuroendocrine differentiation can also be observed during treatment, particularly in the advanced disease stages. t-NEPC has been reported in 10–100% of patients with treatment-resistant mCRPC [3, 8–10]. t-NEPCs are increasingly being recognized as distinct clinical entities, particularly in the context of prolonged exposure to potent ARSIs, which may drive lineage plasticity and progression to AR-independent disease [4]. Although de novo NEPC and t-NEPC share poor prognosis and resistance to AR-directed therapies, they differ in their clinical trajectories. According to a multicenter study by Conteduca et al., the median OS from initial prostate cancer diagnosis was significantly shorter in patients with de novo NEPC (16.8 months) compared to those with t-NEPC (53.5 months). However, no significant OS differences were observed when calculated from the onset of metastatic disease or from the time of NEPC diagnosis [11]. Case reports of NEPC development during triplet therapy are rare. The present case emphasizes the importance of early histological reassessment in patients exhibiting atypical progression, even with initially effective treatments, such as triplet therapy.

Early diagnosis and appropriate treatment of NEPC are essential to improve patient outcomes; however, no specific biomarkers have been firmly established to date [4]. When NEPC is clinically suspected, histopathological evaluation via tissue biopsy remains the gold standard for its diagnosis. NEPC reportedly presents with liver metastases more frequently than conventional CRPC [11], and the NCCN guidelines recommend performing metastatic biopsies when feasible in patients with newly developed visceral metastases during prostate cancer treatment to confirm or exclude t-NEPC [1]. In clinical settings, performing biopsies of metastatic lesions in patients with CRPC can be challenging owing to their poor general condition and technical difficulties, particularly in cases of bone-dominant disease. Nonetheless, when NEPC is clinically suspected based on discordant PSA levels and radiographic findings, early histological reevaluation should be considered.

Given the rarity of NEPC and the lack of established standard therapies, tissues obtained from biopsies should be submitted for CGP when possible. NEPC is characterized by frequent inactivation of tumor suppressor genes, such as RB1, TP53, and PTEN, which have been associated with resistance to AR-targeted therapies [9, 12, 13]. Genomic differences have been observed between de novo and t-NEPC. De novo NEPC reportedly harbors fewer mutations in TP53 and RB1, whereas exhibiting a higher frequency of alterations in DNA damage repair genes such as BRCA1/2 [14]. In contrast, t-NEPC tends to show a higher prevalence of TP53 and RB1 mutations with relatively fewer DNA repair gene alterations [3]. In the present case, comprehensive genomic analysis revealed RB1 loss and PTEN deletion, thereby supporting the molecular phenotype of NEPC. Unfortunately, no actionable genomic alterations have been identified to guide further therapy.

Currently, there is no established standard treatment for NEPC. In clinical practice, platinum-based chemotherapy, typically a combination of cisplatin or carboplatin with etoposide, is often administered according to the protocols used for small cell lung cancer (SCLC) [15]. In our case, this regimen resulted in a transient reduction in the tumor burden and a decline in NSE levels. However, the therapeutic effect was not durable, and rapid disease progression occurred after four cycles. Although immune checkpoint inhibitors (ICIs) have demonstrated efficacy in SCLC and are being explored for NEPC, their clinical benefits in this setting remain limited, and ICIs are not currently recognized as the standard therapy for NEPC [10]. One explanation for the limited efficacy of ICIs in NEPC may lie in the tumor’s biological characteristics: NEPC typically exhibits a low tumor mutational burden and an immunosuppressive tumor microenvironment, both of which may hinder effective immune activation [4, 10, 16]. Further elucidation of the molecular mechanisms underlying NEPC and development of novel therapeutic strategies based on these findings remain urgent clinical priorities.

In the present case, NEPC became clinically apparent shortly after the initiation of triplet therapy, suggesting that neuroendocrine differentiation may have occurred in the early stages of treatment. With the increasing use of potent ARSIs, the incidence of t-NEPC is increasing. As triplet therapy is widely adopted as the standard first-line approach for high-volume mCSPC, the frequency of t-NEPC is expected to increase further. Therefore, clinicians must remain vigilant and consider early histopathological reassessment when atypical clinical features, such as radiological progression or abnormal tumor markers, emerge, even in the context of suppressed PSA levels. Large-scale studies are needed to elucidate the incidence and risk factors of NEPC associated with triplet therapy. Furthermore, accumulating clinical experience, such as early diagnosis and prompt therapeutic adjustments, as demonstrated in this case, is crucial for establishing effective management strategies for NEPC.

## Conclusion

This case highlights the potential for early neuroendocrine differentiation in patients with mCSPC undergoing triple therapy, even in the presence of marked PSA suppression. Clinicians should be vigilant of atypical progression patterns and consider early biopsy of metastatic lesions to ensure an accurate diagnosis and timely therapeutic modification. As the use of intensified treatment strategies has increased, individualized management based on tumor biology has become essential for optimizing patient outcomes.

## Data Availability

No datasets were generated or analyzed during the current study.
